# Transcriptome-Based Six-Gene Fatty Acid Metabolism Signature for Prognosis and Predicted Immunotherapy Response in Lung Adenocarcinoma: Cross-Population Validation

**DOI:** 10.3390/genes17080914

**Published:** 2026-07-31

**Authors:** Qiangping Ma, Jianqing Liang, Jintian Li, Juan Li

**Affiliations:** 1 School of Clinical Medicine of Traditional Chinese Medicine, Gansu University of Chinese Medicine, Lanzhou 730101, China; 2 Key Laboratory of Dunhuang Medicine and Transformation, Ministry of Education, Lanzhou 730000, China; 3 School of Basic Medical Sciences, Gansu University of Chinese Medicine, Lanzhou 730101, China

**Keywords:** lung adenocarcinoma, fatty acid metabolism, elastic net, prognostic signature, immune infiltration, immunotherapy response

## Abstract

Objectives: Lung adenocarcinoma (LUAD) is molecularly heterogeneous, and the prognostic relevance of fatty acid metabolism (FAM) remains incompletely defined. We aimed to develop a concise FAM-associated prognostic signature and examine its associations with the immune microenvironment and candidate therapeutic vulnerabilities. Methods: TCGA-LUAD transcriptomic and survival data were integrated with MSigDB FAM gene sets. Univariate Cox and elastic-net Cox regression were used to derive a risk score. The locked formula was evaluated in a Japanese cohort (GSE31210) and a U.S. cohort (GSE72094). Immune-infiltration algorithms as well as TIDE, GDSC2, and CPTAC data were used for exploratory immune, drug sensitivity, and protein-level analyses. Results: The six-gene signature comprised CYP4B1, ACOXL, DPEP2, HPGDS, CA4, and ALOX15. High-risk patients had shorter overall survival in the TCGA and both external cohorts (GSE31210, log-rank *p* = 0.0039; GSE72094, *p* < 0.0001). The risk score remained independently prognostic after adjustment for age, sex, and clinical stage. High-risk tumours showed lower immune and stromal signals, greater immune exclusion, and a lower TIDE-predicted ICB response proportion. GDSC2 analyses and expression comparisons identified associations with predicted drug sensitivity and lipogenic target expression. Five detectable signature proteins were less abundant in tumours in CPTAC data. Conclusions: This signature stratified patients by prognosis in two geographically distinct external cohorts and generated testable metabolic and immune hypotheses. Prospective validation, assay standardisation, and functional studies are required before clinical use.

## 1. Introduction

Lung cancer is among the most frequently diagnosed cancers and is a leading cause of cancer mortality worldwide [1]. Lung adenocarcinoma (LUAD), the most common histological subtype of non-small-cell lung cancer, accounts for approximately 40–50% of cases and is highly heterogeneous [2]. Targeted therapies and immune checkpoint blockade (ICB) have improved outcomes for selected patients. However, many cases present at an intermediate or advanced stage, and postoperative recurrence and metastasis remain common [3]. Reliable prognostic tools may therefore improve risk stratification and support the precision management of LUAD.

Metabolic reprogramming is a recognised hallmark of malignancy [4]. Tumour cells frequently increase lipogenesis, lipid uptake, and lipid storage to support membrane synthesis and survival under nutrient or oxygen deprivation [5]. Fatty acid metabolism (FAM) also influences signalling, energy storage, and the tumour immune microenvironment [6,7]. Prognostic studies on LUAD have often focused on ferroptosis, glycolysis, or hypoxia [8,9,10]. By contrast, systematic studies that combine FAM-based modelling with cross-population validation and immune or therapeutic analyses remain limited [11].

Elastic-net regression combines L1 and L2 penalties to select variables while retaining information from correlated predictors [12]. This property is relevant to FAM enzymes, which often participate in correlated metabolic programmes. We therefore intersected TCGA-LUAD differentially expressed genes with MSigDB FAM genes and derived an elastic-net Cox signature. We evaluated the locked signature in a Japanese GEO cohort and a U.S. GEO cohort. We then examined its associations with immune composition, TIDE-predicted ICB response, drug sensitivity estimates, and CPTAC protein abundance. Finally, we compared its prognostic performance with glycolysis- and hypoxia-based signatures. This workflow was designed to assess reproducibility and generate experimentally testable metabolic and immune hypotheses.

## 2. Results

### 2.1. Patient Clinical Characteristics and Differentially Expressed Genes

**Figure 1:**
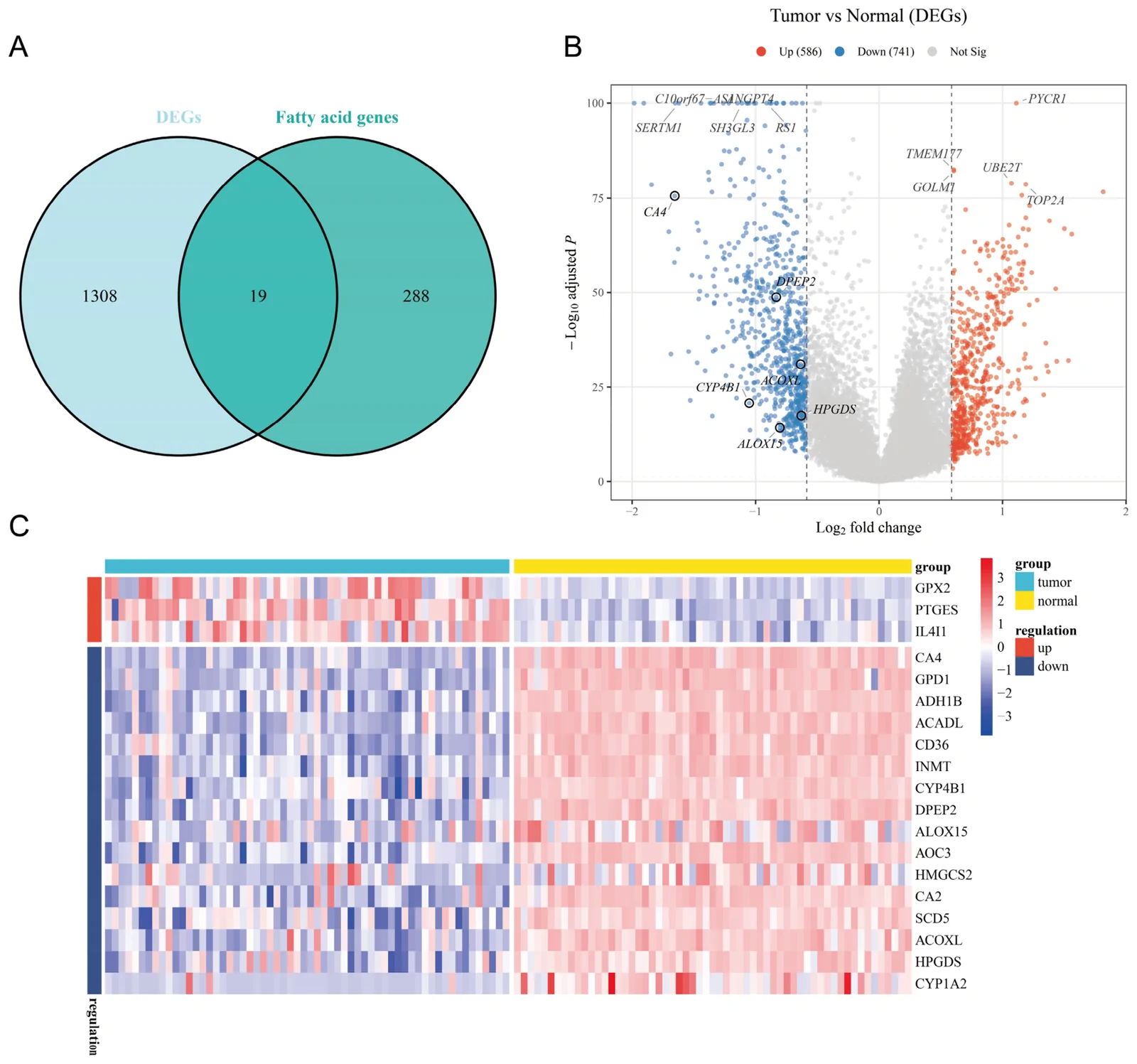
Differential expression analysis and candidate fatty acid metabolism genes. (**A**) Venn diagram of differentially expressed genes and FAM genes, with 19 genes in the intersection as candidates; (**B**) volcano plot of tumour versus adjacent non-tumour differential expression (586 upregulated genes in red, 741 downregulated genes in blue, and nonsignificant genes in grey); six signature genes are circled and labelled. Dashed vertical lines indicate log_2_FC = ±0.585, and dashed horizontal line indicates BH-adjusted *p* = 0.05 (−log_10_ = 1.301). The line appears close to the baseline because the *y*-axis extends to 100; larger values were capped at 100. (**C**) Expression heatmap of 19 candidate genes in tumour and adjacent non-tumour tissues. Tumour and normal samples are indicated with cyan and gold annotation bars, respectively, whereas upregulated and downregulated genes are indicated with red and dark blue side bars, respectively.

After preprocessing, 505 tumour samples with complete survival data and 59 adjacent non-tumour samples were included. Table 1 summarises the baseline characteristics of the high- and low-risk groups. The high-risk group included greater proportions of men and patients with advanced-stage and T or N categories. Differential analysis identified 1327 genes between tumour and adjacent non-tumour tissues. Intersecting these genes with 307 FAM genes yielded 19 candidate FAM genes spanning fatty acid oxidation, synthesis, and metabolic regulation (Figure 1). Differentially expressed genes were defined by |log_2_FC| > 0.585 and BH-adjusted *p* < 0.05. Figure 1B marks the fold-change thresholds at ±0.585 and the statistical threshold at −log_10_(0.05) = 1.301. This horizontal line lies close to the *x*-axis because the ordinate spans 0–100; values above 100 were capped at 100 for visualisation.

### 2.2. Selection of the Six-Gene Prognostic Signature

**Figure 2:**
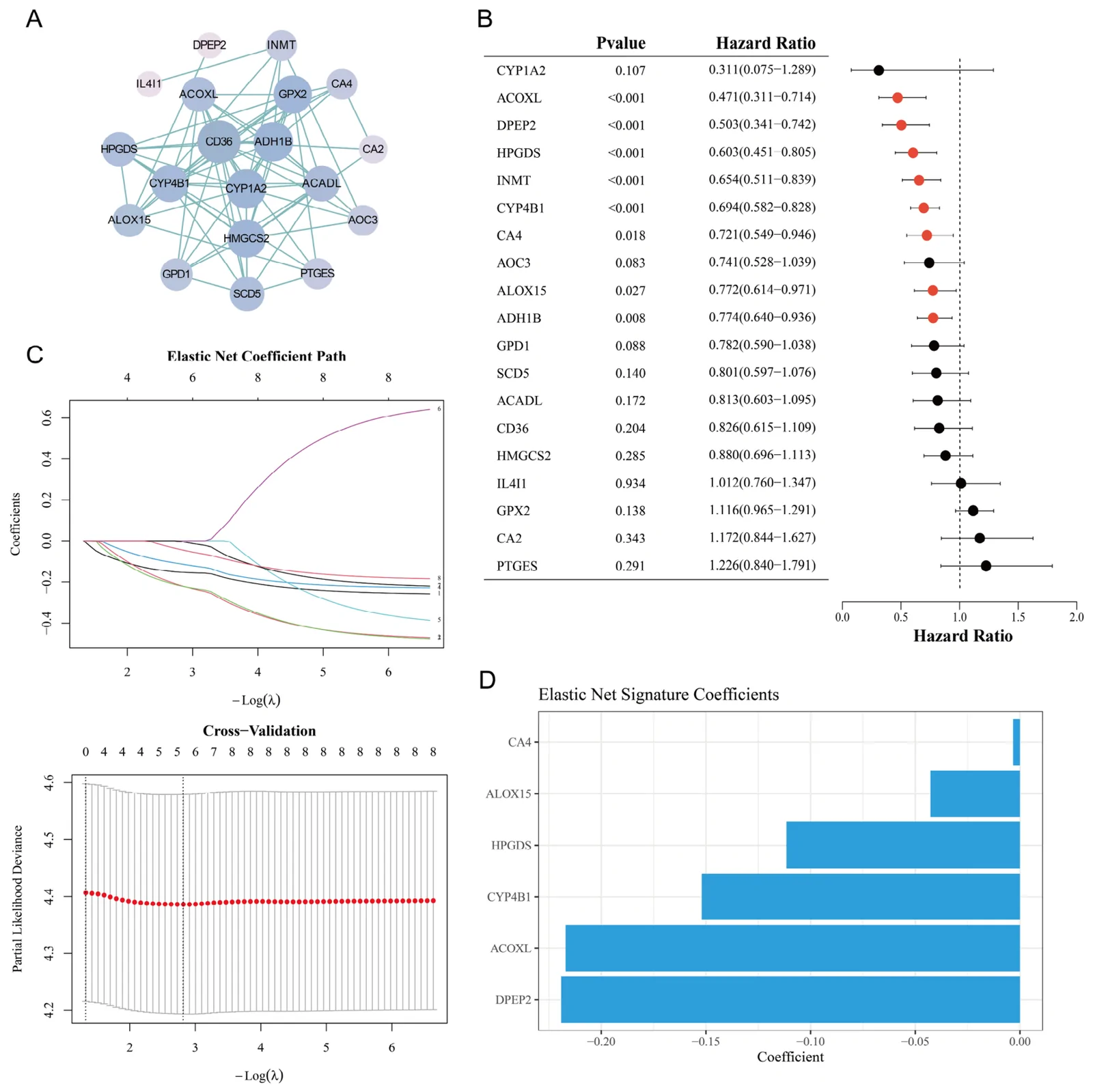
Construction of a fatty acid metabolism-related prognostic signature by elastic-net regression. (**A**) Protein–protein interaction (PPI) network of the 19 candidate genes; (**B**) forest plot of univariate Cox regression for the candidate genes (red, *p* < 0.05; black, *p* ≥ 0.05); (**C**) elastic-net coefficient paths (top; each coloured line represents one candidate gene) and 10-fold cross-validation for λ selection (bottom; the red curve shows mean partial-likelihood deviance and the grey bars show ±1 standard error); (**D**) regression coefficients of the final six-gene signature (all negative). In panel A, the STRING/Cytoscape network comprises 19 genes and 71 undirected associations (*Homo sapiens*; combined score ≥ 0.15): six high-confidence (≥0.70), ten medium-confidence (0.40–0.69), and 55 low-confidence (0.15–0.39) associations. HPGDS centres the high-confidence core, whereas CD36 has the highest degree (15). Node size and fill tone indicate degree, with larger and darker nodes representing higher degree; the uniformly coloured edges denote retained STRING associations and do not encode confidence; STRING scores indicate evidence confidence rather than direct binding strength.

A protein–protein interaction network summarised STRING functional associations among the 19 candidate genes (Figure 2A). To reproduce the connected Cytoscape network shown in the figure, associations with a STRING combined score ≥ 0.15 were retained, yielding 19 nodes and 71 undirected edges: six high-confidence or strong (≥0.70), ten medium-confidence (0.40–0.69), and 55 low-confidence or weaker associations (0.15–0.39). The high-confidence core comprised HPGDS–PTGES (0.958), HPGDS–CYP1A2 (0.954), ALOX15–GPX2 (0.933), ALOX15–CYP1A2 (0.915), HPGDS–GPX2 (0.897), and HPGDS–ADH1B (0.732). Representative medium-confidence links were ACADL–HMGCS2 (0.645), ACADL–CD36 (0.611), and CA2–CA4 (0.557). CD36 had the highest degree (15), whereas ADH1B had the highest betweenness centrality (0.505), followed by CA4 (0.256). All eight ACOXL links were low-confidence, and DPEP2 and IL4I1 each had only one low-confidence link. Thus, the high-confidence core carries the strongest evidence, while the weaker layer provides a connected exploratory overview; STRING scores do not represent direct biophysical binding strength. Univariate Cox analysis identified eight genes associated with overall survival (OS; *p* < 0.05; Figure 2B). Elastic-net Cox regression (α = 0.4) retained CYP4B1, ACOXL, DPEP2, HPGDS, CA4, and ALOX15 (Figure 2C,D). All coefficients were negative. CA4 had the smallest absolute coefficient and was retained as a supporting variable within the correlated feature set. Excluding CA4 did not change the C-index or five-year AUC, and the two risk scores were nearly identical (Spearman’s r = 0.999; Supplementary Table S4). The final risk score formula was:

Risk Score = −0.152 × CYP4B1 − 0.217 × ACOXL − 0.219 × DPEP2 − 0.112 × HPGDS − 0.003 × CA4 − 0.043 × ALOX15

### 2.3. Apparent Performance in TCGA Training Cohort

**Figure 3:**
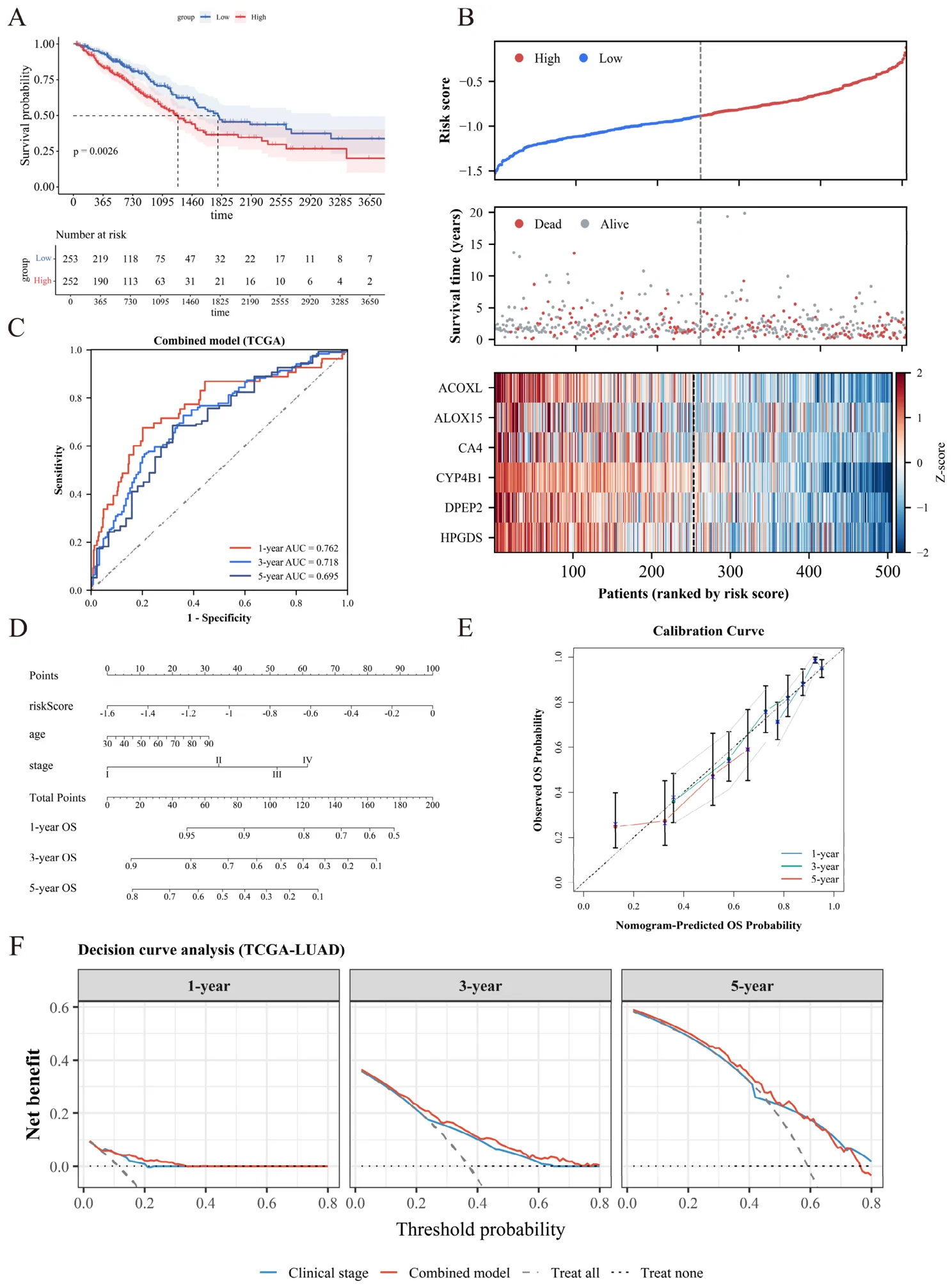
Apparent performance and prognostic nomogram of the model in the TCGA training cohort. (**A**) Kaplan–Meier survival curves for the high- and low-risk groups (log-rank *p* = 0.0026; with number-at-risk table and censoring marks); (**B**) risk triplot (from top to bottom: ranked risk score, survival status scatter plot [red, death], and signature gene expression heatmap); (**C**) time-dependent ROC curves of the combined model at one, three and five years; (**D**) prognostic nomogram integrating the risk score, age and clinical stage; (**E**) calibration curves for one-, three- and five-year overall survival (blue, one year; red, three years; green, five years; grey 45° dashed line, perfect calibration); (**F**) decision curve analysis at one, three and five years comparing the combined model, clinical stage, and treat-all/treat-none strategies.

Using the training set median, 252 patients were assigned to the high-risk group and 253 to the low-risk group. OS was shorter in the high-risk group (log-rank *p* = 0.0026; Figure 3A). The one-, three-, and five-year AUCs for the signature were 0.684, 0.632, and 0.621, respectively. The risk triplot showed more deaths at higher risk scores and generally lower expression of the six genes (Figure 3B). The corresponding AUCs for the combined clinical model were 0.762, 0.718, and 0.695 (Figure 3C). These estimates describe apparent performance in the modelling cohort and may be optimistic. Generalisability was therefore assessed in two external GEO cohorts.

### 2.4. Cross-Population External Validation in Two Cohorts

**Figure 4:**
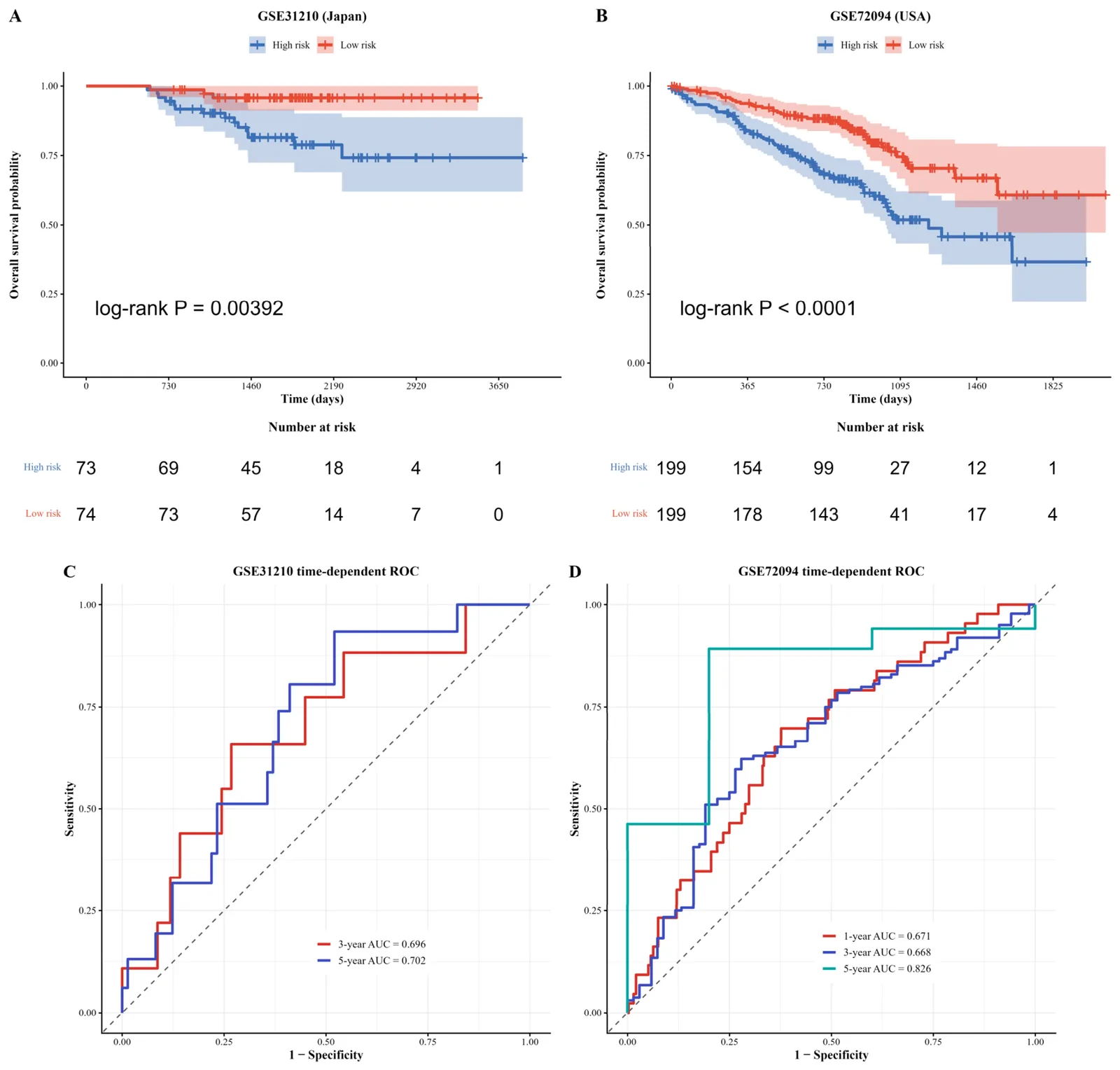
Cross-population external validation in two cohorts. (**A**,**B**) Kaplan–Meier survival curves for the high- and low-risk groups in GSE31210 (Japan) and GSE72094 (USA), respectively; (**C**,**D**) time-dependent ROC curves in GSE31210 and GSE72094, respectively. Log-rank *p* values were 0.0039 and <0.0001 after cohort-specific preprocessing. Time-dependent AUCs were 0.696/0.702 (three/five years) for GSE31210 and 0.671/0.668/0.826 (one/three/five years) for GSE72094.

The locked six-gene coefficients were evaluated independently in the two GEO cohorts after cohort-specific preprocessing. GSE31210-MAS5-normalised intensities were transformed using log_2_(x + 1). Among 147 patients with complete survival data, OS was shorter in the high-risk group (log-rank *p* = 0.0039; HR = 5.19, 95% CI 1.49–18.07; Figure 4A). Because this predominantly early-stage cohort had few deaths within one year, only the three- and five-year AUCs were estimated (0.696 and 0.702; Figure 4C). For GSE72094, the author-provided IRON-normalised and RNA quality PLS-corrected matrix was used. OS again differed between risk groups (log-rank *p* < 0.0001; HR = 2.29, 95% CI 1.56–3.38; Figure 4B). The one-, three-, and five-year AUCs were 0.671, 0.668, and 0.826, respectively (Figure 4D). Few patients remained at risk at five years, so the final estimate should be interpreted cautiously. These findings support performance in Japanese and United States cohorts but do not establish universal population transportability (Supplementary Table S5).

### 2.5. Independent Prognostic Analysis and Incremental Value

The risk score was associated with OS in univariate analysis (HR = 4.188, 95% CI 2.403–7.300; *p* < 0.001). It remained independently prognostic after adjustment for age, sex, and clinical stage (HR = 3.613, 95% CI 2.00–6.53; *p* < 0.001). The combined model had a C-index of 0.698 (95% CI 0.653–0.747), compared with 0.665 for stage alone. Its one-, three-, and five-year AUCs were 0.762, 0.718, and 0.695, respectively (Supplementary Table S6). Thus, the signature provided incremental prognostic information within the TCGA rather than replacing clinical staging. The integrated nomogram is shown in Figure 3D. Calibration curves compare predicted and observed outcomes (Figure 3E), and decision curve analysis compares the combined model with stage-only, treat-all, and treat-none strategies (Figure 3F).

### 2.6. Differences in Immune Microenvironment Between Risk Groups

**Figure 5:**
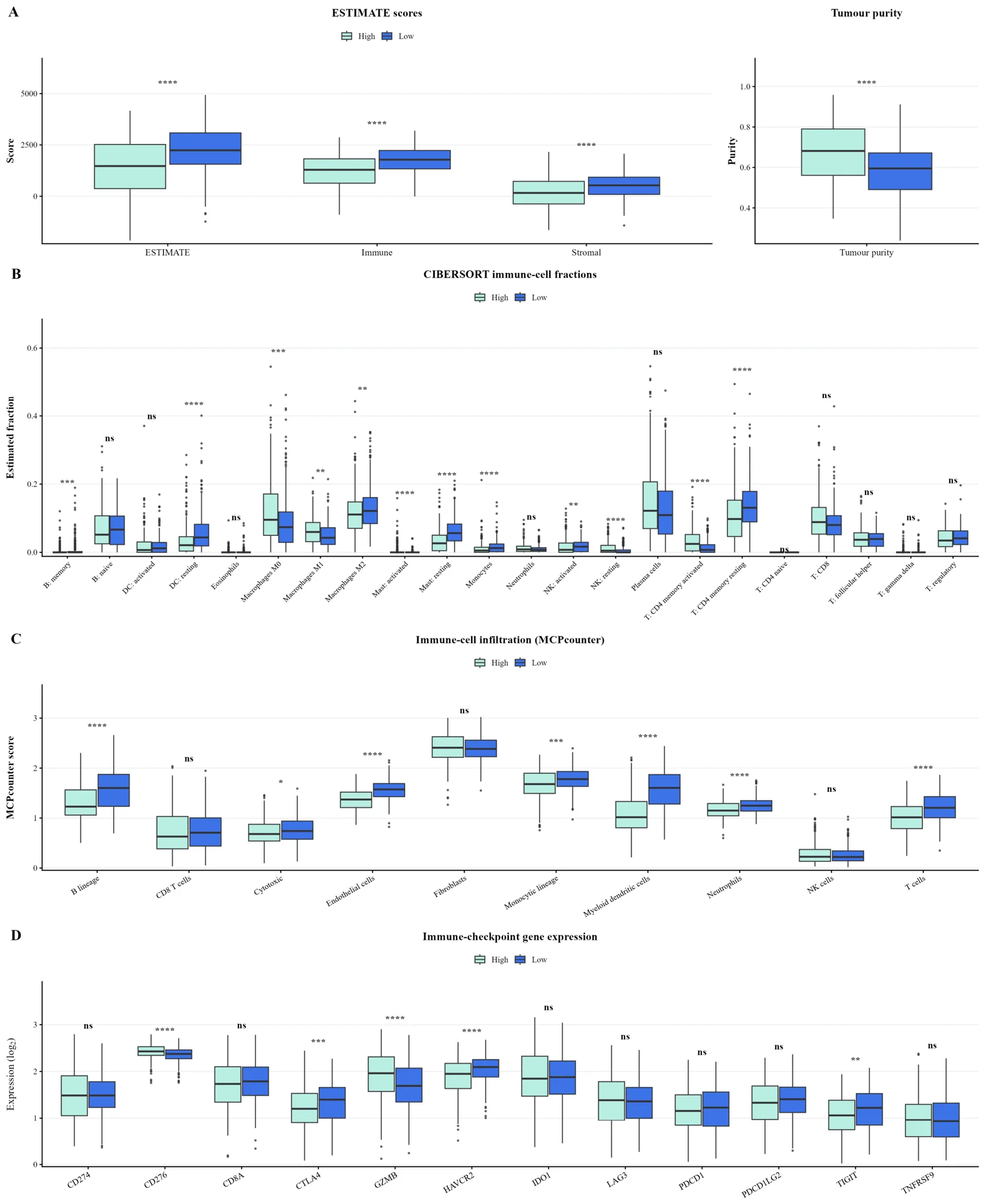
Differences in the immune microenvironment between the high- and low-risk groups. (**A**) ESTIMATE stromal, immune and combined scores and tumour purity; (**B**) fractions of 22 immune-cell types estimated by CIBERSORT; (**C**) immune and stromal cell signals estimated by MCPcounter; (**D**) expression of selected immune-checkpoint genes. Key findings were lower stromal, immune and ESTIMATE scores and higher tumour purity in the high-risk group. MCPcounter showed significantly lower B-lineage, cytotoxic-lymphocyte, endothelial, monocytic-lineage, myeloid-dendritic, neutrophil and total T-cell signals; CD8 T-cell score was lower descriptively but nonsignificant after BH correction. Among the checkpoint genes, CD276 and GZMB were higher, whereas CTLA4, HAVCR2 and TIGIT were lower, in the high-risk group. Stars above each comparison denote BH-adjusted two-sided Wilcoxon rank-sum *p* values within the corresponding panel (ns, adjusted *p* ≥ 0.05; * adjusted *p* < 0.05; ** adjusted *p* < 0.01; *** adjusted *p* < 0.001; **** adjusted *p* < 0.0001).

IOBR analyses identified broad differences in the immune microenvironment between risk groups. Wilcoxon *p* values are BH-adjusted within each method panel in Figure 5. The high-risk group had lower immune, stromal, and ESTIMATE scores and higher tumour purity (all adjusted *p* < 0.0001; Figure 5A). A total of 12 of 22 CIBERSORT fractions remained significant after correction (Figure 5B). The high-risk group had lower memory B-cell, resting memory CD4 T-cell, monocyte, M2-macrophage, resting dendritic-cell and resting mast-cell fractions. Activated memory CD4 T-cell and M0/M1-macrophage fractions were higher. MCPcounter showed lower total T-cell, cytotoxic-lymphocyte, B-lineage, monocytic-lineage, myeloid-dendritic-cell, neutrophil and endothelial-cell signals (Figure 5C). The CD8 T-cell estimate was lower but nonsignificant after correction. CTLA4, HAVCR2, and TIGIT expression was lower, whereas CD276 and GZMB expression was higher (Figure 5D). CD274, PDCD1, and LAG3 did not differ. Together, these findings are consistent with a broadly immune-depleted expression pattern in the high-risk group. Correlations between the risk score and CIBERSORT immune-cell fractions, together with complementary xCell comparisons, are presented in Supplementary Figure S1.

### 2.7. Immunogenicity and Immune Landscape

We analysed the number of non-silently mutated genes per sample as a proxy for tumour mutational burden (TMB) and compared pan-cancer immune subtypes. This mutation count proxy was higher in the high-risk group (median 246 vs. 105 genes; *p* < 0.001). It also correlated positively with the risk score (Spearman r = 0.40; *p* < 0.001). The immune subtype distribution differed between groups (chi-squared *p* < 0.001). C1 and C2 predominated in the high-risk group, whereas C3 was more frequent in the low-risk group. Supplementary Figure S2 presents these analyses.

### 2.8. Functional Enrichment Analysis

Functional enrichment was performed using 182 differentially expressed genes between the risk groups. GO over-representation analysis identified 97 significant terms, including humoral immune response, chromosome segregation, cilium movement, and cell killing. KEGG analysis identified cell cycle, DNA replication, mismatch repair, p53 signalling, arachidonic acid metabolism, and linoleic acid metabolism (Supplementary Table S2). GSEA showed enrichment of cell-cycle and DNA-repair pathways in the high-risk group. The low-risk group was enriched in cilium movement, humoral immunity, cell adhesion, and lipid metabolism pathways (Supplementary Figure S3). Supplementary Table S1 compares this signature with glycolysis- and hypoxia-based signatures.

### 2.9. Prediction of Immunotherapy Response and Drug Sensitivity

**Figure 6:**
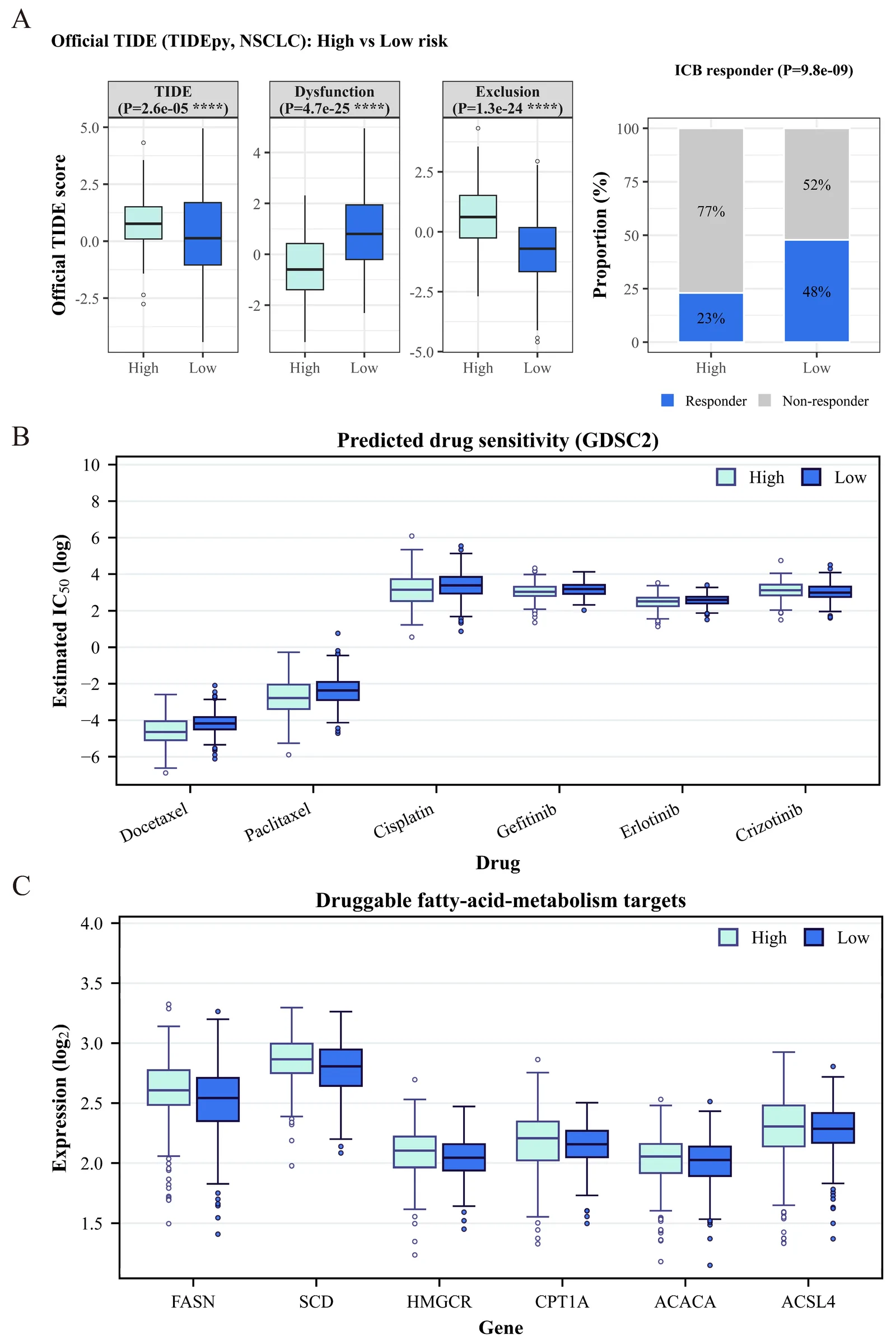
Prediction of immunotherapy response and drug sensitivity. (**A**) Official TIDE scores (composite TIDE, Exclusion and Dysfunction) and predicted ICB responder proportion in high- and low-risk groups; (**B**) GDSC2-predicted IC_50_ values for chemotherapeutic and targeted agents; (**C**) expression of druggable fatty acid metabolism targets (FASN, SCD, HMGCR, CPT1A, ACACA and ACSL4). Key computational findings were higher composite TIDE and Exclusion scores, lower Dysfunction scores and a lower predicted ICB responder proportion in the high-risk group (23.0% vs. 47.8%); lower predicted IC_50_ values for docetaxel and paclitaxel; and significantly higher expression of FASN, SCD and HMGCR. Asterisks indicate significance (****, *p* < 0.0001).

The official TIDE NSCLC model was used to estimate the ICB response. The high-risk group had higher composite TIDE (*p* = 2.6 × 10^−5^), immune-exclusion (*p* = 1.3 × 10^−24^), and MDSC scores (*p* < 0.001). Its predicted responder proportion was lower (23.0% vs. 47.8%; χ^2^ *p* = 9.8 × 10^−9^; Figure 6A). The low-risk group had a higher dysfunction score (*p* = 4.7 × 10^−25^) but lower composite TIDE and exclusion scores. In GDSC2-based analyses, seven of ten agents differed between groups. The predicted IC_50_ values were lower in the high-risk group for docetaxel (*p* = 1.59 × 10^−11^) and paclitaxel (*p* = 2.03 × 10^−6^). Representative agents are shown in Figure 6B, with complete results in Supplementary Table S3. EGFR-TKI estimates should be interpreted with respect to EGFR mutation status. SCD (*p* = 6.6 × 10^−5^), HMGCR (*p* = 1.2 × 10^−3^), and FASN (*p* = 2.1 × 10^−3^) were more highly expressed in the high-risk group (Figure 6C). These computational associations prioritise therapeutic hypotheses but do not establish treatment eligibility or clinical efficacy.

### 2.10. Protein-Level Validation of Signature Genes

**Figure 7:**
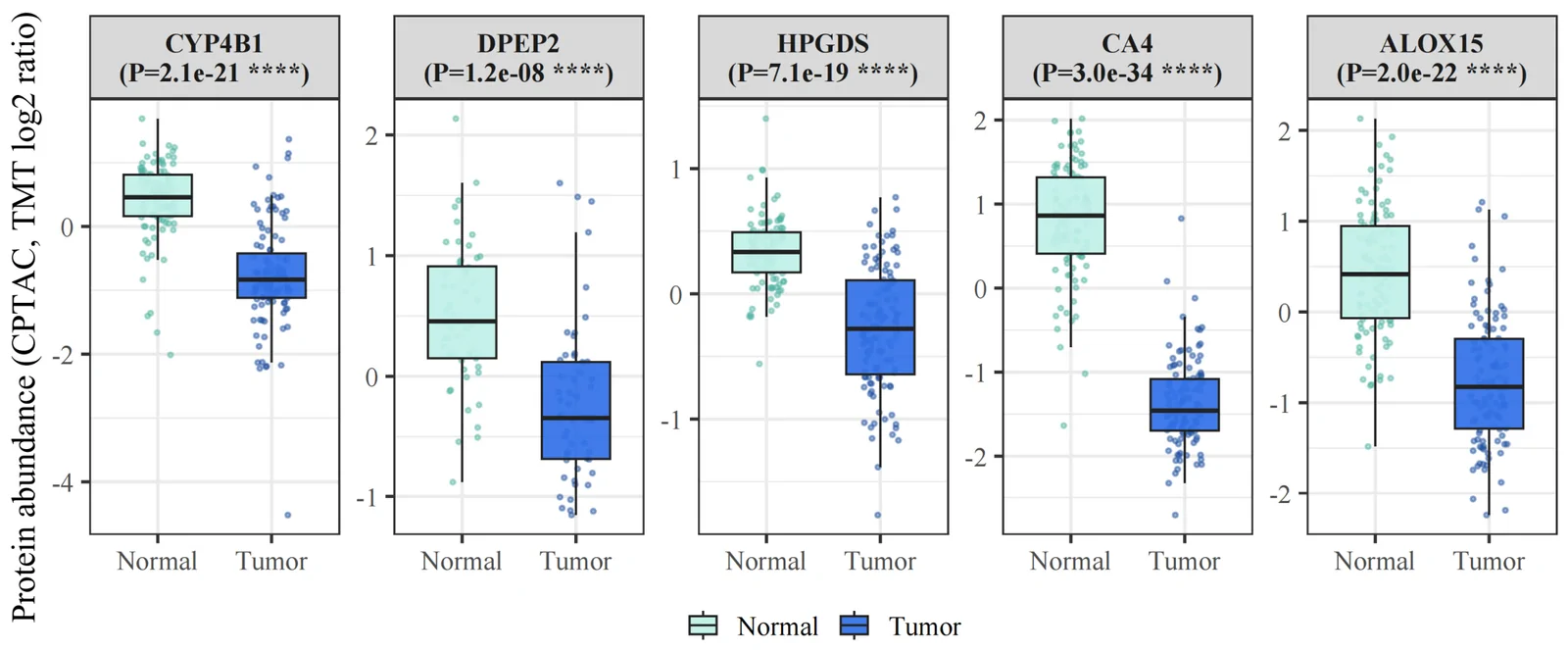
Tumour versus adjacent non-tumour protein abundance of signature genes in the CPTAC cohort. Box plots show CPTAC protein abundance (TMT log_2_ ratio) for five mass-spectrometry-detected signature genes (CYP4B1, DPEP2, HPGDS, CA4, and ALOX15); ACOXL was not detected. *p* values were calculated using Wilcoxon rank-sum test (****, *p* < 0.0001).

We compared tumour and adjacent non-tumour protein abundance using the CPTAC LUAD proteome accessed through cProSite [2]. ACOXL was not detected by mass spectrometry. The other five proteins, CYP4B1, DPEP2, HPGDS, CA4, and ALOX15, were less abundant in tumours (all *p* < 0.0001; Figure 7). This direction was concordant with their transcript-level differences and negative model coefficients. The CPTAC analysis supports cross-platform consistency but does not independently validate the prognostic performance of the six-gene score.

## 3. Discussion

We derived a six-gene FAM-associated prognostic signature and evaluated the locked formula in GSE31210 and GSE72094. The risk score was independently prognostic in the TCGA, and the combined model provided greater discrimination than stage alone. Concordant survival separation in Japanese and United States cohorts is a central strength of the study. Elastic net was suitable because its combined L1/L2 penalty can retain information from correlated metabolic genes. The six genes span fatty acid oxidation and eicosanoid-related processes, although their inclusion does not establish pathway causality. Prior work has linked glycolysis-associated LUAD signatures to the tumour microenvironment [13]. In our head-to-head comparison, glycolysis- and hypoxia-based signatures showed stronger discrimination (Supplementary Table S1). The present signature should therefore be viewed as a complementary FAM-associated model rather than a superior general metabolic predictor.

Importantly, validation on GSE31210 showed that the signature retained prognostic value in a Japanese cohort (after MAS5 normalisation and log_2_(x + 1) transformation, log-rank *p* = 0.0039; three- and five-year time-dependent AUCs of 0.696 and 0.702, respectively), providing evidence of performance in a non-Caucasian East Asian population. This cross-population result is an important step toward reducing population-related bias in LUAD prognostic modelling. Nevertheless, validation in a single Japanese cohort does not establish universal transportability, and prospective evaluation in additional Asian, African, Hispanic/Latino and other under-represented populations is warranted.

All six coefficients were negative, indicating that lower expression was associated with higher estimated risk. CYP4B1 participates in fatty acid ω-hydroxylation and is downregulated in several solid tumours [14]. ALOX15 participates in lipid peroxidation and has shown tumour-suppressive activity [15], whereas HPGDS is linked to pulmonary immune homeostasis [16]. CA4 requires more cautious interpretation. It is a carbonic anhydrase included in the MSigDB HALLMARK fatty acid metabolism set, with an indirect metabolic link through bicarbonate supply. Its near-zero coefficient and unchanged five-gene sensitivity results indicate minimal incremental discrimination. CA4 is therefore classified as a supporting variable and prospective biomarker, not a core FAM-specific effector. It was retained because it belonged to the prespecified candidate set and remained in the correlated feature group selected at λ.min. This model selection result should not be interpreted as evidence of FAM specificity. Five detected signature proteins were less abundant in CPTAC tumours, supporting concordant dysregulation across molecular layers.

The immune analyses link the signature to distinct tumour microenvironment states. The high-risk group showed lower immune and stromal scores and broad reductions in lymphoid and dendritic-cell signals. These results support an immune-desert-like pattern, although individual algorithms did not agree for every cell type. The mutation count proxy was higher in the high-risk group, yet TIDE predicted greater immune exclusion and a lower responder proportion. This apparent discordance may reflect the incomplete translation of mutation-associated antigenicity into effective immunity. The low-risk group had higher T-cell dysfunction scores but lower composite TIDE and exclusion scores. Thus, infiltration and dysfunction should be interpreted jointly rather than as interchangeable measures [17]. These findings are consistent with earlier FAM score studies [11,18] and reports linking LUAD stromal states to immunotherapy resistance [19]. Recent studies also connect mitochondrial lipid metabolism and fatty acid oxidation with LUAD immune states and osimertinib resistance [20,21]. Nevertheless, the present immune results remain computational associations.

TIDE is a transcriptome-based prediction framework and does not reproduce the full set of determinants of clinical ICB benefit. RECIST-defined response in LUAD is also influenced by directly measured TMB, PD-L1 protein expression, EGFR or ALK alterations, co-mutations, treatment line, and host factors. Here, the number of non-silently mutated genes was only a TMB proxy and is not equivalent to panel- or exome-derived mutations per megabase. The predicted response difference is therefore hypothesis-generating. It requires validation in independent ICB-treated LUAD cohorts with RECIST 1.1 response and survival outcomes. Such analyses should jointly model direct TMB, PD-L1, driver alterations, and relevant clinical covariates.

The drug analyses generated additional hypotheses for high-risk tumours. GDSC2 models predicted lower IC_50_ values for docetaxel and paclitaxel, while FASN, SCD, and HMGCR expression was higher. Published FASN inhibitors, acetyl-CoA carboxylase inhibitors, and statin-repurposing strategies provide experimental starting points [22,23,24]. However, the six-gene score did not directly measure target dependence, and no patient-level treatment interaction was tested. The model’s translational value currently lies in prioritising biomarker-stratified experiments. Its small gene set may also facilitate future assay development using quantitative PCR or immunohistochemistry [25], subject to analytical validation and calibration.

GDSC2-derived IC_50_ estimates and target expression differences do not demonstrate clinical efficacy. Cell lines do not reproduce intratumoural heterogeneity, stromal or immune interactions, drug exposure, toxicity, or prior treatment. A staged strategy could first test FASN or SCD inhibitors and statins in organoids derived from patients with LUAD classified by the six-gene score. Relevant endpoints would include viability, lipid flux, and target engagement. Matched patient-derived xenografts or humanised PDX models could then assess tumour response, pharmacokinetics, and toxicity. Positive findings would justify biomarker-stratified prospective studies. Until then, these agents should be considered experimental candidates rather than treatment recommendations.

This study has several limitations. First, all cohorts were retrospective, and prospective validation was lacking. Second, each validation cohort was dichotomised by its own median risk score. This approach tests within-cohort stratification but does not define a portable clinical cutoff across assay platforms. Third, the early-stage GSE31210 cohort had too few one-year events, and few GSE72094 patients remained at risk at five years. These time-specific AUCs should therefore be interpreted cautiously. Fourth, the TIDE and GDSC2 analyses are computational and require validation in clinically annotated ICB cohorts, organoids, and PDX models. Prospective studies should predefine the assay, normalisation procedure, risk threshold, and clinical endpoints.

In summary, a six-gene FAM-associated signature stratified OS in TCGA-LUAD and two geographically distinct external cohorts. The signature was also associated with immune composition, a mutation count proxy, TIDE predictions, drug sensitivity estimates, and CPTAC protein abundance. These results support cross-population prognostic relevance and generate testable metabolic and immune hypotheses. Prospective, functional, and assay standardisation studies are required before the signature can inform clinical decisions.

## 4. Methods

### 4.1. Data Sources

TCGA-LUAD STAR-FPKM expression, clinical and survival data were downloaded from UCSC Xena (589 samples: 530 tumours and 59 adjacent non-tumour samples). We deduplicated the HALLMARK, KEGG and REACTOME FAM gene sets from MSigDB to obtain 307 genes [26]. External validation datasets GSE31210 (GPL570, Japan; 54,675 probes across 246 deposited samples) and GSE72094 (GPL15048, USA; 60,607 probes across 442 deposited samples) were obtained from GEO. GEO metadata reported MAS5 normalisation for GSE31210 and IRON normalisation followed by partial least squares correction for RNA quality effects for GSE72094. In this study, GSE31210 intensities were transformed as log_2_(x + 1), whereas the deposited GSE72094 values were used without an additional log transformation. The cohorts were processed independently; no pooled normalisation or cross-cohort ComBat correction was applied. GDSC2 data included 805 cell lines and 198 drugs [27]. Tumour and adjacent non-tumour CPTAC LUAD protein abundance data were accessed through cProSite [2]. All data were publicly available.

### 4.2. Data Pre-Processing and Differential Expression Analysis

Tumour and adjacent non-tumour samples were identified from TCGA barcodes. Ensembl identifiers were mapped to gene symbols, and duplicate mappings were averaged. Cases with missing survival time or survival shorter than 30 days were excluded, leaving 505 tumours for modelling. Expression values were transformed as log_2_(FPKM + 1). Differential expression was analysed using limma with |log_2_FC| > 0.585 and BH-adjusted *p* < 0.05. Differentially expressed genes were intersected with the 307 FAM genes. The 19 candidates were submitted to the STRING network endpoint for *H. sapiens* (species = 9606; required_score = 150). Duplicate pairs were removed, and unique functional associations with a combined score ≥ 0.15 were retained [28], yielding 71 undirected edges connecting all 19 candidates. The edge table and Cytoscape node metrics table were imported into Cytoscape 3.10.4. Network degree was defined as the number of undirected edges incident on each node, and node size and fill tone were scaled to degree for visualisation. Betweenness centrality was obtained using Cytoscape’s Analyze Network function. Combined scores of 0.15–0.39, 0.40–0.69, and ≥0.70 were classified as low-confidence, medium-confidence, and high-confidence/strong associations, respectively. The low-confidence layer was retained to show the complete connected topology, while confidence stratification was used to distinguish exploratory links from the high-confidence core. STRING scores indicate database evidence confidence rather than direct biophysical binding strength.

### 4.3. Construction of Prognostic Signature

Candidate genes associated with OS in univariate Cox regression (*p* < 0.05) were modelled using elastic-net Cox regression in glmnet. We used α = 0.4 and selected λ.min by 10-fold cross-validation with set.seed(123). Risk score was calculated as Σ(β_i_ × Expr_i_), and patients in the TCGA were divided at the training set median. The α value was selected to balance sparsity with retention of correlated FAM predictors. In a sensitivity grid with identical folds across α = 0.1–1.0, α = 0.2–0.4 selected the same six genes. At α = 0.4, the minimum mean cross-validated partial-likelihood deviance was within 0.0053 of the best grid result. We therefore retained α = 0.4 as a stable compromise, not as a uniquely optimal value.

### 4.4. Model Evaluation and External Validation

OS was compared using Kaplan–Meier curves and the log-rank test. Time-dependent AUCs were estimated at one, three and five years using timeROC. C-index 95% CIs were obtained from 1000 bootstrap resamples. The two GEO cohorts were analysed separately, without pooled normalisation or ComBat correction. GSE31210 MAS5 intensities were transformed as log_2_(x + 1), and 147 cases with non-missing survival time and status were retained. GSE72094 IRON-normalised and RNA quality PLS-corrected values were used as deposited, without further log transformation; 398 cases with non-missing positive survival time and status were retained. GPL570 and GPL15048 probes were mapped to official gene symbols, and multiple probes per gene were averaged. Risk scores were calculated using the locked TCGA coefficients, with no coefficient refitting in either validation cohort. Because absolute expression scales differed between platforms, groups were defined using the median within each validation cohort. This design evaluates within-cohort stratification but does not establish a common clinical cutoff.

### 4.5. Independent Prognostic Analysis

The risk score, age, sex, and clinical stage were assessed in univariate and multivariable Cox models. TCGA clinical stage followed the annotated AJCC, seventh edition. C-indices and time-dependent AUCs were compared among stage-only, signature-only, and combined models to assess incremental prognostic information.

### 4.6. Immune Microenvironment and Immunotherapy Response Analysis

IOBR integrated ESTIMATE, MCPcounter, CIBERSORT, ssGSEA, and xCell estimates of immune or stromal composition and tumour purity [29]. We also compared CD274, PDCD1, CTLA4, LAG3, HAVCR2, TIGIT, and other checkpoint-related genes. TCGA-LUAD somatic mutations were obtained from the MC3 non-silent gene-level matrix in UCSC Xena. The number of non-silently mutated genes per sample was used as a mutation burden proxy, not standard mutations-per-megabase TMB. Pan-cancer immune subtypes were assigned according to Thorsson et al. [30]. TIDEpy was run with the NSCLC model on mean-centred expression profiles. Composite TIDE, Dysfunction, Exclusion, MDSC, and predicted response outputs were compared between risk groups [31].

### 4.7. Drug Sensitivity Analysis and Functional Enrichment

Drug sensitivity was explored using two complementary strategies. Ridge regression models (glmnet, α = 0) were trained on GDSC2 expression and IC_50_ values to estimate sensitivity to ten selected agents in TCGA samples. Expression of the six candidate fatty acid metabolism targets FASN [22], SCD, HMGCR, CPT1A, ACACA, and ACSL4 was also compared. GO and KEGG over-representation analyses used differentially expressed genes between the risk groups. GSEA was performed with clusterProfiler using all genes ranked by log_2_FC [32]. Glycolysis- and hypoxia-based signatures were constructed using the same workflow for comparison (Supplementary Table S1).

### 4.8. Statistical Analysis

Analyses were performed in R 4.4.3 using limma 3.62.2, survival 3.8-3, glmnet 4.1-10, survminer 0.5.2, timeROC 0.4.1, rms 8.1-1, GEOquery 2.74.0, IOBR 2.2.3.9000, GSVA 2.0.7, and clusterProfiler 4.14.6. TIDE analyses used TIDEpy 1.3.9. Continuous variables were compared using the Wilcoxon rank-sum test and categorical variables using the chi-squared test. Fisher’s exact test was used when expected frequencies were small. Survival differences were assessed using the log-rank test. Cox models reported hazard ratios and 95% CIs, and correlations used Spearman coefficients. Unless stated otherwise, tests were two-sided, and *p* < 0.05 indicated statistical significance. For multi-feature comparisons, *p* values were adjusted by the Benjamini–Hochberg procedure within the relevant analysis panel, as specified.

## Figures and Tables

**Table 1:** Clinical baseline characteristics of high- and low-risk groups among 505 patients with LUAD [*n* (%)].

Characteristic	High-Risk Group (*n* = 252)	Low-Risk Group (*n* = 253)	*p* Value
Age ≤ 65 years	135 (53.6)	107 (42.3)	0.017
Age > 65 years	113 (44.8)	140 (55.3)	
Male	138 (54.8)	96 (37.9)	<0.001
Female	114 (45.2)	157 (62.1)	
Clinical stage I	115 (45.6)	160 (63.2)	<0.001
Clinical stage II	71 (28.2)	47 (18.6)	
Clinical stage III	49 (19.4)	30 (11.9)	
Clinical stage IV	14 (5.6)	11 (4.3)	
T1–T2	213 (84.5)	227 (89.7)	0.077
T3–T4	38 (15.1)	24 (9.5)	
N0	151 (59.9)	177 (70.0)	0.003
N1–N3	99 (39.3)	65 (25.7)	
Distant metastasis (M1)	14 (5.6)	10 (4.0)	0.673

## Data Availability

All data analysed in this study are publicly available. TCGA-LUAD transcriptomic, clinical, survival, and mutation data are available through UCSC Xena (https://xenabrowser.net/; accessed on 24 July 2026). GSE31210 and GSE72094 are available from the Gene Expression Omnibus (https://www.ncbi.nlm.nih.gov/geo/; accessed on 24 July 2026). CPTAC LUAD proteomic data were accessed through cProSite (https://cprosite.ccr.cancer.gov/; accessed on 24 July 2026). Fatty acid metabolism gene sets are available from MSigDB (https://www.gsea-msigdb.org/; accessed on 24 July 2026). GDSC2 data are described by the Wellcome Sanger Institute GDSC resource (https://www.sanger.ac.uk/tool/gdsc-genomics-drug-sensitivity-cancer/; accessed on 24 July 2026). Analysis parameters are described in the Section 4 and Supplementary Materials.

## Supplementary Materials

The following supporting information can be downloaded at: https://www.mdpi.com/article/10.3390/genes17080914/s1. Figure S1: Association between the risk score and immune infiltration. (A) Correlation between the risk score and CIBERSORT immune-cell fractions (lollipop plot); (B) immune cell types differing significantly between the high- and low-risk groups, estimated by xCell; Figure S2: Immunogenicity and immune landscape. (A) Box plot of the mutational-burden proxy (number of non-silently mutated genes per sample, used as a proxy for tumour mutational burden; y-axis log10-transformed) in the high- and low-risk groups; (B) scatter plot of the correlation between the risk score and this mutational-burden proxy; (C) distribution of the six pan-cancer immune subtypes (C1–C6) by risk group; Figure S3: Functional enrichment analysis. (A) GO enrichment bubble plot of the differentially expressed genes (BP/CC/MF); (B,C) GSEA lollipop plots (B, GO; C, KEGG; red, activated in the high-risk group; blue, suppressed in the low-risk group); Table S1: Head-to-head comparison of metabolic-pathway signatures in TCGA-LUAD; Table S2: GO and KEGG enrichment of differentially expressed genes between the high- and low-risk groups (representative terms; 97 GO and 66 KEGG terms, all adjusted *p* < 0.05); Table S3: Predicted IC50 values of 10 GDSC2 agents between the high- and low-risk groups (seven with *p* < 0.05); Table S4: Sensitivity analysis of the five-gene model excluding CA4 (TCGA-LUAD training cohort); Table S5: Model performance of the signature in the training and two cross-population validation cohorts; Table S6: Predictive performance of different models in the TCGA-LUAD training cohort.
