# Supplementary material for: Transcriptome-Based Six-Gene Fatty Acid Metabolism Signature for Prognosis and Predicted Immunotherapy Response in Lung Adenocarcinoma: Cross-Population Validation
Source: Genes (Basel). 2026 Jul 31;17(8):914. doi: 10.3390/genes17080914 (PMC13511999; doi:10.3390/genes17080914)
Supplement: Supplementary file 1 [file genes-17-00914-s001.zip › genes-4449260-supplementary.pdf]

Supplementary Information

Transcriptome-Based Six-Gene Fatty Acid Metabolism Signature for Prognosis and Predicted Immunotherapy Response in Lung Adenocarcinoma: Cross-Population Validation

Supplementary Figures

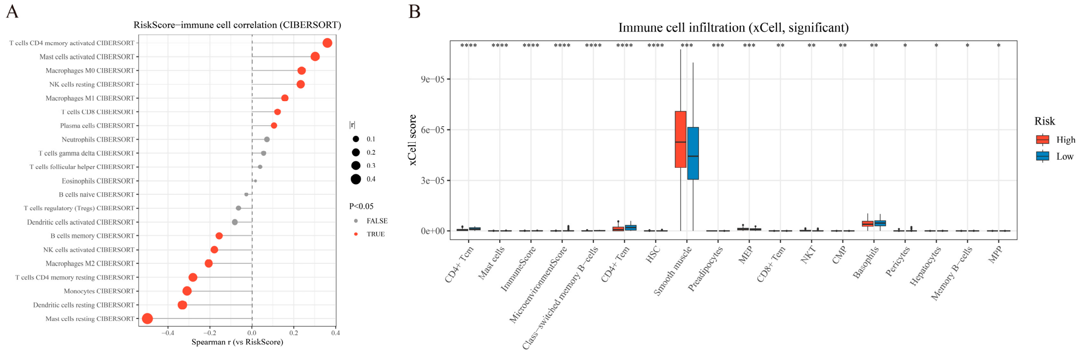

Figure S1. Association between the risk score and immune infiltration. A, correlation between the risk score and CIBERSORT immune-cell fractions (lollipop plot); B, immune cell types differing significantly between the high- and low-risk groups, estimated by xCell.

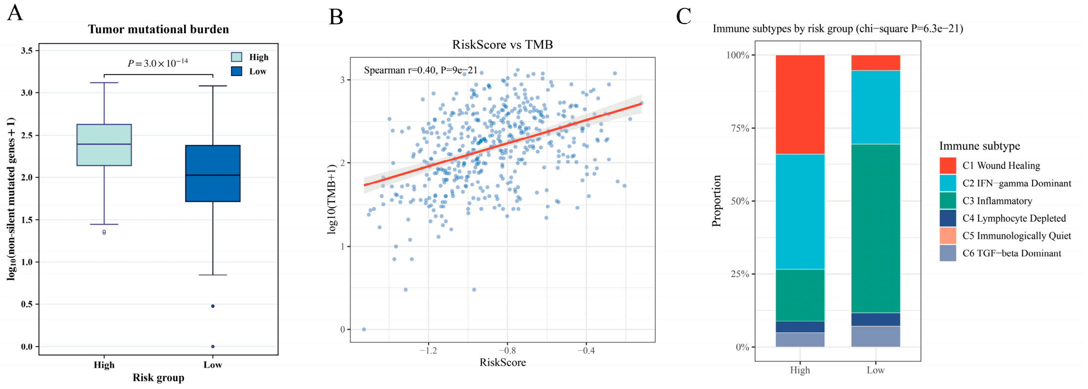

Figure S2. Immunogenicity and immune landscape. A, box plot of the mutational-burden proxy (number of non-silently mutated genes per sample, used as a proxy for tumour mutational burden, TMB; y-axis log10-transformed) in the high- and low-risk groups; B, scatter plot of the correlation between the risk score and this mutational-burden proxy; C, distribution of the six pan-cancer immune subtypes (C1–C6) by risk group.

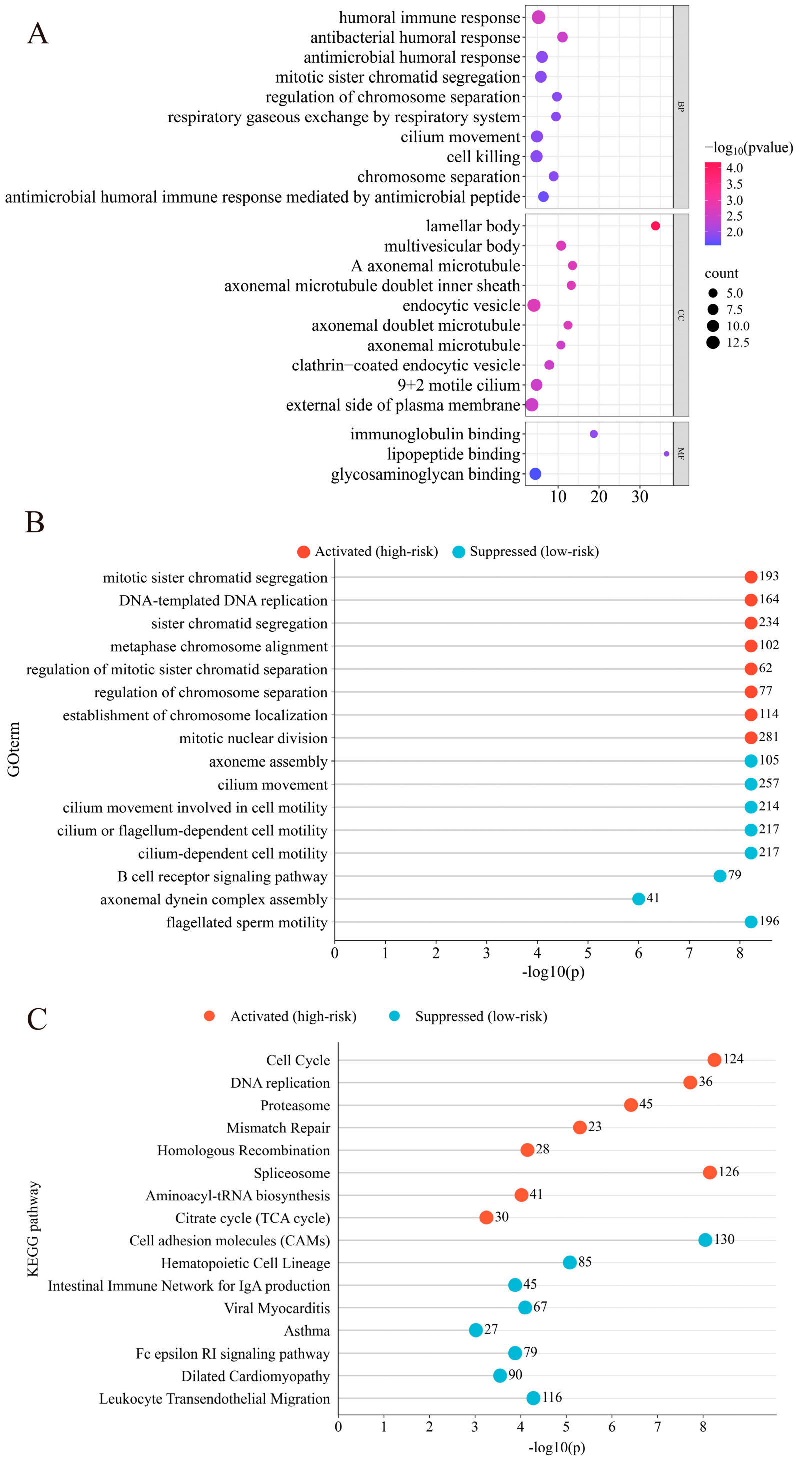

Figure S3. Functional enrichment analysis. A, GO enrichment bubble plot of the differentially expressed genes (BP/CC/MF); B and C, GSEA lollipop plots (B, GO; C, KEGG; red, activated in the high-risk group; blue, suppressed in the low-risk group).

Supplementary Tables

Supplementary Table S1. Head-to-head comparison of metabolic-pathway signatures in TCGA-LUAD

Pathway signature	No. of genes	C-index	5-year AUC
Glycolysis signature (HALLMARK_GLYCOLYSIS)	14	0.717	0.711
Hypoxia signature (HALLMARK_HYPOXIA)	12	0.706	0.673
Fatty acid metabolism signature (this study)	6	0.622	0.621
FAM signature + clinical model (this study)	6 + 2	0.698	0.695

Note: Glycolysis- and hypoxia-based signatures showed stronger discrimination in the TCGA-LUAD training cohort. The present fatty acid metabolism signature provides complementary prognostic information. When combined with clinical features, it yielded a C-index of 0.698 with six genes. Associations with candidate lipid-metabolism targets remain exploratory and require experimental validation.

Supplementary Table S2. GO and KEGG enrichment of differentially expressed genes between the high- and low-risk groups (representative terms; 97 GO and 66 KEGG terms, all P.adj < 0.05)

Type	Term	Statistic	P.adj
GO·BP	humoral immune response	Count = 13	2.8×10^{-3}
GO·BP	cell killing	Count = 10	1.4×10^{-2}
GO·BP	cilium movement	Count = 10	1.3×10^{-2}
GO·BP	sister chromatid segregation	Count = 9	1.3×10^{-2}
GO·CC	lamellar body	Count = 5	6.8×10^{-5}
GO·CC	endocytic vesicle	Count = 12	1.9×10^{-3}
KEGG ↑ (high risk)	cell cycle	NES = +2.55	9.3×10^{-9}
KEGG ↑ (high risk)	DNA replication	NES = +2.39	2.9×10^{-8}
KEGG ↑ (high risk)	mismatch repair	NES = +2.26	4.4×10^{-6}
KEGG ↑ (high risk)	homologous recombination	NES = +2.21	4.4×10^{-5}
KEGG ↑ (high risk)	p53 signalling pathway	NES = +1.64	3.0×10^{-3}
KEGG ↓ (low risk)	arachidonic acid metabolism	NES = −1.66	7.3×10^{-4}
KEGG ↓ (low risk)	linoleic acid metabolism	NES = −1.46	4.2×10^{-2}

KEGG ↓ (low risk)	cell adhesion molecules	NES = −1.82	2.3×10^{-8}

Supplementary Table S3. Predicted IC_{50} of 10 GDSC2 agents between the high- and low-risk groups (7 with P < 0.05)

Drug (GDSC ID)	High-risk $\log_{10}(IC_{50})$	Low-risk $\log_{10}(IC_{50})$	P value	More sensitive group
Docetaxel (GDSC 1007)	−4.60	−4.17	1.6×10^{-11}	High risk
Docetaxel (GDSC 1819)	−2.31	−1.72	5.3×10^{-7}	High risk
Paclitaxel (GDSC 1080)	−2.78	−2.38	2.0×10^{-6}	High risk
Gefitinib (GDSC 1010)	3.06	3.19	1.3×10^{-4}	High risk
Erlotinib (GDSC 1168)	2.47	2.59	2.5×10^{-4}	High risk
Cisplatin (GDSC 1005)	3.16	3.39	1.4×10^{-3}	High risk
Crizotinib (GDSC 1083)	3.12	3.02	4.5×10^{-3}	Low risk
Osimertinib (GDSC 1919)	1.68	1.63	0.58 (NS)	—
Gemcitabine (GDSC 1190)	−0.47	−0.46	0.81 (NS)	—
Pyridostatin (GDSC 2044)	3.41	3.43	0.92 (NS)	—

Supplementary Table S4. Sensitivity analysis of the five-gene model excluding CA4 (TCGA-LUAD training cohort)

Model	C-index	1-year AUC	3-year AUC	5-year AUC	Log-rank P
6-gene (incl. CA4)	0.622	0.684	0.632	0.621	0.0026
5-gene (excl. CA4)	0.622	0.684	0.631	0.621	0.0026

Note: Spearman's r between the two risk scores was 0.999; after removing the near-zero-coefficient gene CA4 (coefficient −0.003), the C-index, time-dependent AUC and log-rank significance were essentially unchanged, confirming that the marginal contribution of CA4 is negligible. The C-index was estimated by coxph concordance.

Supplementary Table S5. **Model performance of the signature in the training and two cross-population validation cohorts**

Cohort	Samples/deaths	Log-rank P	1-year AUC	3-year AUC	5-year AUC	C-index
TCGA-LUAD (training)	505/181	0.0026	0.684	0.632	0.621	0.622
GSE31210 (Japan, validation 1)	147/17	0.0039	NA	0.696	0.702	0.696
GSE72094 (USA, validation 2)	398/113	<0.0001	0.671	0.668	0.826	0.642

Note: this table reports the predictive performance of the signature (risk score) across the three cohorts (only the signature was validated, as the external cohorts lacked harmonised clinical variables). For GSE31210, the 1-year AUC could not be estimated (NA) owing to too few deaths within one year.

Supplementary Table S6. Predictive performance of different models in the TCGA-LUAD training cohort

Model	C-index	1-year AUC	3-year AUC	5-year AUC
Clinical stage only	0.665	—	—	—
Signature only	0.622	0.684	0.632	0.621
Signature + clinical combined	0.698	0.762	0.718	0.695
